# Shear Thickening Fluid and Sponge-Hybrid Triboelectric Nanogenerator for a Motion Sensor Array-Based Lying State Detection System

**DOI:** 10.3390/ma17143536

**Published:** 2024-07-17

**Authors:** Youngsu Kim, Inkyum Kim, Maesoon Im, Daewon Kim

**Affiliations:** 1 Department of Electronics and Information Convergence Engineering, Institute for Wearable Convergence Electronics, Kyung Hee University, 1732 Deogyeong-daero, Giheung-gu, Yongin 17104, Republic of Korea; 2 Brain Science Institute, Korea Institute of Science and Technology (KIST), Seoul 02792, Republic of Korea; 3 Division of Bio-Medical Science and Technology, KIST School, University of Science and Technology (UST), Seoul 02792, Republic of Korea; 4 KHU-KIST Department of Converging Science and Technology, Kyung Hee University, Seoul 02447, Republic of Korea; 5 Department of Electronic Engineering, Institute for Wearable Convergence Electronics, Kyung Hee University, 1732 Deogyeong-daero, Giheung-gu, Yongin 17104, Republic of Korea; 6 Center for BrainTechnology, Korea Institute of Science and Technology, 5, Hwarang-ro 14-gil, Seongbuk-gu, Seoul 02792, Republic of Korea

**Keywords:** triboelectric nanogenerator, shear thickening fluid, sponge, motion sensor array, lying state detection system

## Abstract

Issues of size and power consumption in IoT devices can be addressed through triboelectricity-driven energy harvesting technology, which generates electrical signals without external power sources or batteries. This technology significantly reduces the complexity of devices, enhances installation flexibility, and minimizes power consumption. By utilizing shear thickening fluid (STF), which exhibits variable viscosity upon external impact, the sensitivity of triboelectric nanogenerator (TENG)-based sensors can be adjusted. For this study, the highest electrical outputs of STF and sponge-hybrid TENG (SSH-TENG) devices under various input forces and frequencies were generated with an open-circuit voltage (*V*_OC_) of 98 V and a short-circuit current (*I*_SC_) of 4.5 µA. The maximum power density was confirmed to be 0.853 mW/m^2^ at a load resistance of 30 MΩ. Additionally, a lying state detection system for use in medical settings was implemented using SSH-TENG as a hybrid triboelectric motion sensor (HTMS). Each unit of a 3 × 2 HTMS array, connected to a half-wave rectifier and 1 MΩ parallel resistor, was interfaced with an MCU. Real-time detection of the patient’s condition through the HTMS array could enable the early identification of hazardous situations and alerts. The proposed HTMS continuously monitors the patient’s movements, promptly identifying areas prone to pressure ulcers, thus effectively contributing to pressure ulcer prevention.

## 1. Introduction

In contemporary society, the utilization of video-based monitoring technology is prevalent across a diverse range of industries, including security, healthcare, and transportation, as evidenced by recent studies [1,2,3]. Despite its widespread adoption, this technology encounters significant challenges, primarily due to its dependency on adequate lighting conditions for optimal operation and increasing concerns regarding privacy violations stemming from pervasive video surveillance [4]. These limitations emphasize the necessity for alternative monitoring approaches that can operate independently of lighting conditions and can mitigate privacy issues. In this context, there is escalating interest in the development and application of electric signal-based monitoring technologies [5]. Among these, triboelectric nanogenerator (TENG) technology emerges as a particularly promising solution. TENGs generate high-voltage electrical signals through the mechanical interactions of contact and separation processes, exploiting the principle of contact electrification and electrostatic induction [6,7]. This technology not only enables the precise determination of sensor positioning but also facilitates the detection of force magnitude, based on the nature of the physical interactions [8,9,10,11]. TENGs offer the distinct advantage of producing electrical outputs autonomously, thereby circumventing the requirement for external power sources or batteries. This attribute notably simplifies device architecture by negating the necessity for ancillary power conversion circuitry [12,13,14]. Moreover, the inherent properties of TENGs allow for a reduction in the size of the devices, which, in turn, enhances the flexibility of their installation. This size reduction, coupled with the elimination of complex power systems, leads to a decrease in power consumption, thereby significantly enhancing the overall efficiency and sustainability of monitoring systems [15,16,17]. Consequently, TENG technology presents a viable and efficient alternative to traditional video-based monitoring systems, promising advancements in both performance and privacy standards.

The operational mechanism of TENGs is fundamentally based on the electrical output generated through the contact-separation dynamics between two distinct materials, wherein the properties of these materials critically influence the electrical output [18]. This attribute is a cornerstone of TENG technology, highlighting the role of material selection. The efficacy of TENGs largely hinges on the ability of the chosen materials to alter their contact area in response to external forces, a phenomenon that plays a crucial role in the functional optimization of these devices. In pursuit of enhancing the sensitivity of TENG-based sensors, the application of shear thickening fluid (STF) has emerged as a notable innovation. STF is characterized by its unique property of undergoing a significant increase in viscosity under specific stress conditions [19,20,21,22,23,24,25]. This behavior is particularly beneficial for TENG applications as the fluid’s heightened viscosity under stress maximizes the frictional forces during the contact and separation phases, thereby amplifying the generated electrical signals. Such an enhancement in frictional interaction not only improves the electrical signal output but also significantly boosts the sensitivity and stability of the sensors, facilitating more precise and reliable monitoring. The formulation of STF involves a mixture of micro-scale powders and liquids, with combinations such as cornstarch and deionized (DI) water serving as a classic example. This mixture adapts well to the operational demands of TENGs by optimizing the material properties to enhance energy conversion efficiency during mechanical interactions. Nonetheless, the deployment of STF in sensor technologies is not devoid of challenges. For instance, the susceptibility of STF materials to environmental conditions such as humidity and nutrients that promote fungal growth necessitates additional considerations. These challenges have been effectively addressed by incorporating sodium propionate into the STF formulation, which acts as a fungicide to inhibit the growth of fungi, thereby preserving the integrity and functionality of STF-TENG systems over extended periods [26,27]. The incorporation of fungicidal agents into TENG-based sensors enhances their long-term stability and operational reliability, representing a considerable leap forward in sensor technology through the reduction of potential degradation and maintenance concerns.

To optimize the generated electrical outputs in TENGs, the engineering of sensor components designed to facilitate the process of contact and separation is imperative [28,29,30,31,32,33,34]. A critical aspect of this design involves the structural configuration of the sensor materials to ensure rapid contact and subsequent separation upon the application of an external force. In this context, a porous structure composed of polydimethylsiloxane (PDMS) has been developed, which effectively forms void spaces that expedite the separation phase following force application, thereby enhancing the dynamic response of the sensor [35,36]. Moreover, to further augment the electrical output of these sensors, the porous PDMS structure has been integrated with polyvinylidene fluoride (PVDF), which possesses inherent piezoelectric properties [37]. This integration leverages the piezoelectric effect, whereby the mechanical stress induced by contact-separation in the sensor structure results in the generation of an electrical charge. The inclusion of PVDF not only amplifies the electrical output but also significantly boosts the sensitivity of the resultant PVDF-PDMS sponge-based (PPS) triboelectric sensor. This enhancement is particularly beneficial in applications requiring high sensitivity and rapid responsiveness, as it ensures that even minimal mechanical interactions are efficiently converted into measurable electrical signals. This strategic combination of material properties and structural design in TENG sensors, as exemplified by the PDMS and PVDF composite, represents a forward step in the development of advanced sensory devices. By optimizing both the material composition and the structural attributes, these sensors can achieve superior performance metrics, rendering them highly suitable for a range of applications where accurate and efficient energy harvesting and motion sensing are crucial.

In this study, the optimization of PVDF concentration within a PDMS sponge-based TENG (PPS-TENG) was explored as a combination yielding the highest electrical output. Moreover, in the development of the STF and sponge-hybrid TENG (SSH-TENG), superior electrical outputs were achieved under specific mechanical stimulations while varying the applied force and frequency. Further investigations into the output power characteristics revealed the maximum power density, which was reached by connecting the external load resistance. This finding emphasizes the potential of these configurations in energy harvesting applications under varied mechanical conditions. The principal objective of this study is to advance the development of a hybrid triboelectric motion sensor (HTMS) array that is specifically tailored for real-time monitoring in medical settings. These sensors are ingeniously designed to detect patient movements with high accuracy, enabling timely interventions in critical situations where patients may remain overly stationary or may face a heightened risk of falls [9,36]. Additionally, the continuous monitoring capability of these sensors plays a crucial role in the early detection and prevention of pressure ulcers in vulnerable areas, providing a proactive approach to healthcare management. This dual functionality not only enhances patient safety but also contributes to the overall efficacy of medical care protocols.

## 2. Materials and Methods

### 2.1. Materials

Polydimethylsiloxane (PDMS) (Dow Silicones Corporation, Sylgard 184, Midland, MI, USA) and polyvinylidene fluoride (PVDF) (Sigma-Aldrich, St. Louis, MA, USA) were purchased.

### 2.2. Demonstrating the Lying State Detection System

The Arduino Nano processes the output signals from the 6 analog channels connected to the HTMS array with Arduino coding. Python coding was conducted to visualize the pressing–releasing state of a hybrid triboelectric motion sensor (HTMS) and to activate the alarm buttons. The packages of PyQt5, pyserial, and matplotlib in Python 3.11.9 were adopted to prepare the application.

### 2.3. Materials Characterization

A tabletop scanning electron microscope (SEM) (COXEM EM-30AXN, Daejeon, Republic of Korea) was used to measure the surface state and analyze the energy dispersive X-ray spectroscopy (EDS) of the PVDF-PDMS sponge; 12 kV and SE (secondary electron) conditions were adopted in the measurement. An optical microscope (OM) (Nikon Corporation Eclipse LV100, Tokyo, Japan) with a 100× objective lens and a 3-megapixel digital camera was used to check the surface of the sugar-PVDF. A Fourier-transform infrared spectrometer (FT-IR) (Bruker ALPHA II, Billerica, MA, USA) was used to check the molecular vibrations and stretching in the PVDF-PDMS sponge.

### 2.4. Output Measurement

Force was exerted using an electrodynamic shaker (Labworks Inc. LW139.138-40, Costa Mesa, CA, USA), regulated by a signal from a function generator (Agilent Technologies Inc. 33120A, Santa Clara, CA, USA), operating under varying input forces and frequencies. The electrical output data were captured using a system electrometer (Keithley Model 6514, Cleveland, OH, USA) interfaced with a multi-channel DAQ system (NI PCI-6220, Austin, TX, USA). A force sensor (Dytran Instruments Inc. 1053v4, Chatsworth, CA, USA) and an amplifier (Dytran Instruments Inc. E4110C, Chatsworth, CA, USA) were used to measure and visualize the applied force using an oscilloscope (Tektronix MSO44 4-BW-200, Beaverton, OR, USA).

### 2.5. Simulation

The finite element method (FEM) was employed to visualize the surface charge profile of the TENGs using COMSOL Multiphysics 5.0 (COMSOL Inc., Stockholm, Sweden).

## 3. Results and Discussion

### 3.1. Preparation of the SSH-TENG

**Figure 1:**
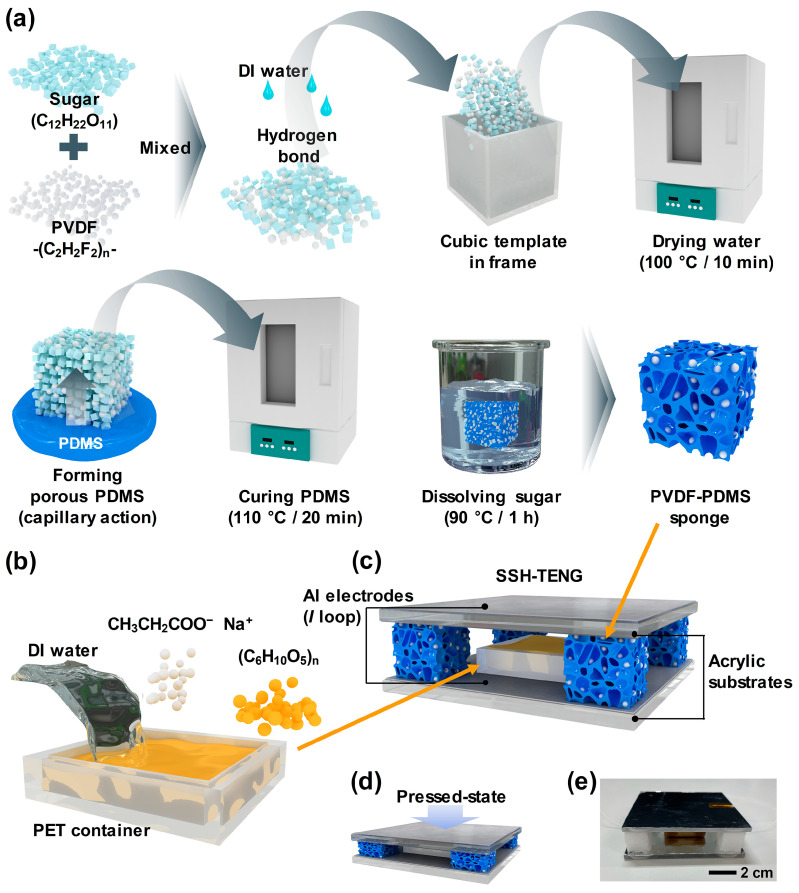
(**a**) Overall schematic illustration of the fabrication process for a PVDF-PDMS sponge (PPS). (**b**) Schematic illustration of shear thickening fluid (STF) layer fabrication. (**c**) Shear thickening fluid and sponge-hybrid TENG (SSH-TENG) illustration image. (**d**) Pressed state of SSH-TENG. (**e**) Digital camera image of SSH-TENG.

Figure 1a illustrates the fabricating process of PVDF-PDMS sponge-based TENG (PPS-TENG). To prepare the PPS, powdered sugar and powdered PVDF were mixed to a total mass of the mixture of 3.5 g. Then, 0.2 mL of water was added using a pipette to utilize hydrogen bonding in fabricating a cubic template of the hydrophilic sugar and hydrophobic PVDF. Sugar molecules contain hydroxyl groups (–OH), which can form hydrogen bonds with water molecules. This principle was employed to mix sugar, PVDF, and water in a 1.5 cm × 1.5 cm × 1.5 cm PET mold. The mixture was then placed in a convection oven and heated at 100 °C for 10 min to shape it back into a cubic template.

The template was placed on top of the uncured PDMS solution for 12 h to fabricate a porous structure through capillary action. Subsequently, the cubes, containing a mixture of sugar, PVDF, and PDMS, were placed in the convection oven and cured at 110 °C for 20 min. After the curing process, the mixture was then immersed in DI water in a beaker and heated to 90 °C for 2 h to dissolve the sugar with magnetic stirring. The PPS fabrication process was finalized, following an overnight drying phase at ambient room temperature.

Figure 1b depicts the fabrication process of the STF (shear thickening fluid) layer. Using PET film, a mold with the dimensions of 3 cm × 3 cm × 0.6 cm was prepared. Then, 3.2 g of DI water and sodium propionate powder were mixed in the mold, followed by the addition of 4.8 g of cornstarch to form the STF substance.

Figure 1c presents a 3D image of the fabricated SSH-TENG (STF and sponge-hybrid TENG), while Figure 1d shows an image of the SSH-TENG in contact with an external force. A digital camera image of the fabricated SSH-TENG is displayed in Figure 1e.

### 3.2. Analysis of the Material Properties

**Figure 2:**
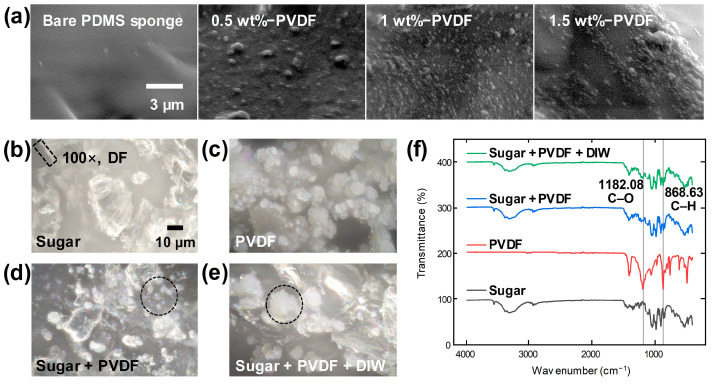
(**a**) SEM image of PPS with PVDF powder in PDMS. Optical microscopy images of (**b**) sugar powder, (**c**) PVDF powder, (**d**) sugar + PVDF powder, and (**e**) sugar + PVDF powder with added DI water. (**f**) FT-IR spectra of sugar powder, PVDF powder, sugar + PVDF powder, and sugar + PVDF powder with added DI water.

Figure 2a shows the surface SEM images of the PVDF-PDMS sponge, with the different wt.% of the PVDF powder with the sugar template. With the increased wt.% of the PVDF to 1 wt.%, the PVDF powders were uniformly inset within the PDMS network. The sponge with over 1 wt.% of PVDF showed agglomeration between the PVDF particles. This aggregation is primarily due to the high electronegativity of fluorine atoms in PVDF, which significantly polarizes the C–F bonds in the PVDF molecules. This results in pronounced polar interactions with the carbon atoms of sugar (C_12_H_22_O_11_), leading to conspicuous bonding. The addition of DI water likely facilitated aggregation through weak hydrogen bonding between PVDF molecules. In the sponge fabricating process, the aggregated PVDF reduces the capillary forces, thereby decreasing the rigidity of the fabricated sponge.

The optical microscopy (OM) images provided in Figure 2b–e offer a detailed visualization of the materials encapsulated within a cubic template, employing a dark field illumination technique to enhance the contrast and visibility of the particle boundaries at a magnification of 100×. Specifically, Figure 2b shows the sugar powder, wherein the edges of each particle are distinctly marked by dotted lines to emphasize their unique, angular morphology. This distinct boundary delineation aids in assessing the particle’s physical properties and spatial distribution. In Figure 2c, the image captures the PVDF powder, characterized by smoother, more uniformly circular boundaries compared to the sugar powder. This image also reveals the presence of numerous smaller particles, each less than 10 µm in diameter, scattered across the surface of the sample, indicating a fine granularity that might influence the bulk properties of the material.

Transitioning to Figure 2d, the OM result of a simple stirring mix of sugar and PVDF powder is depicted. Here, the sharp-edged sugar particles are clearly separated from the smoother PVDF particles. This separation highlights the distinct physical characteristics of each material and suggests minimal interaction or blending at the macroscopic level under gentle mixing conditions. Furthermore, the image identifies those areas where PVDF particles are distributed in small-sized clusters, circled with dotted lines to indicate their localized distribution. Finally, Figure 2e demonstrates the morphological changes upon adding DI water to the sugar and PVDF mixture. The process leads to slight dissolution and the subsequent drying of the sugar, which results in a unique interaction where PVDF powders are found adhering to the altered sugar particles. This interaction potentially modifies the surface characteristics and the mechanical properties of the particles. Within the dotted circles, the aggregation of PVDF particles can be observed, which may be critical in understanding the behavior of composite material under different environmental conditions. This comprehensive analysis through OM images not only elucidates the individual and collective particle behaviors but also provides insights into the potential applications and performance of these materials in practical settings.

The FT-IR spectroscopy data presented in Figure 2f can be used to critically examine the chemical interactions and bonding characteristics of sugar and PVDF, as well as between the PVDF molecules themselves. The analysis specifically focuses on the influence of adding deionized (DI) water to the mixture of sugar and PVDF. The FT-IR spectra reveal significant enhancements in specific vibrational modes indicative of molecular interactions. Notably, the peak corresponding to the C–O stretching vibration at 1182.08 cm^−1^ is more pronounced in the spectrum of the sugar + PVDF + DI water mixture compared to the sugar + PVDF mixture without DI water. This enhancement suggests increased interaction between the sugar and PVDF molecules, potentially facilitated by the polar nature of the water molecules. Additionally, the C–H bending vibration at 868.63 cm^−1^ shows a similar enhancement upon the addition of DI water. This observation supports the hypothesis that hydrogen bonding, introduced by the water, significantly strengthens the interactions not only between sugar molecules but also between PVDF molecules. This hydrogen bonding likely leads to more stable and complex molecular arrangements within the composite material.

Both spectra from the sugar + PVDF and sugar + PVDF + DI water mixtures display mixed states of peaks, which are characteristic of both sugar and PVDF. This blending of spectral features indicates a composite material where the molecular structures of sugar and PVDF are intermingled, possibly leading to novel material properties. The insights derived from these FT-IR spectra emphasize the crucial role of water as a facilitator of enhanced chemical bonding in sugar-PVDF composites. By mediating stronger and more numerous hydrogen bonds, DI water not only influences the physical properties of the composite but also potentially enhances its application in areas where improved material interaction is beneficial.

**Figure 3:**
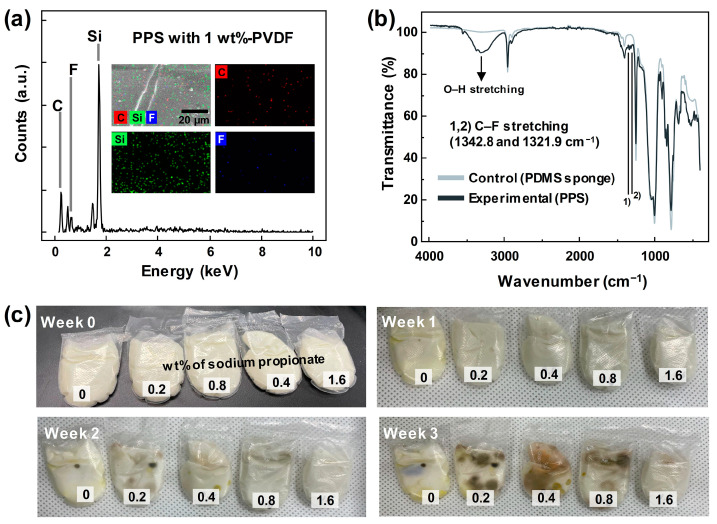
(**a**) EDX result and elemental mapping results. (**b**) FT-IR spectra of bare PDMS and PPS. (**c**) Fungus-checking results in the STF with different concentrations of sodium propionate.

The PVDF at the surface of the PDMS sponge can be checked with the EDS and elemental mapping results in Figure 3a and FT-IR spectra in Figure 3b. The fluorine atom can be checked with both the EDS result and the distributed blue dots in the mapping result. By comparing the FT-IR spectra of the bare PDMS sponge and PVDF-PDMS sponge, it can be seen that the small peaks at the 1342.8 and 1321.9 cm^−1^ indicate the C–F stretching in the PVDF, not in the PDMS.

Figure 3c displays images of fungus growth according to the wt.% of sodium propionate. DI water and cornstarch, as representative materials of STF, provide a suitable environment for fungus growth due to their moist conditions and protein provision as energy sources. Sodium propionate, an acidic compound, inhibits fungal growth by inducing an acidic environment. To demonstrate this suppression, STF was placed inside a polyethylene bag, and sodium propionate was added at concentrations of 0.2, 0.4, 0.8, and 1.6 wt.%. Observation revealed that fungus formed in the STF without sodium propionate after 1 week, while fungus formation was observed in the 0.2, 0.4, and 0.8 wt.% samples after 2 weeks. However, fungus growth was inhibited in the 1.6 wt.% sample for up to 2 weeks. Starting from 3 weeks, fungus was observed even with the 1.6 wt.% sample, but the area of fungus growth was significantly smaller compared to the 0.2, 0.4, and 0.8 wt.% samples, demonstrating that fungus inhibition varied with the wt.% of sodium propionate.

### 3.3. Working Principle and Simulation Results of SSH-TENG

**Figure 4:**
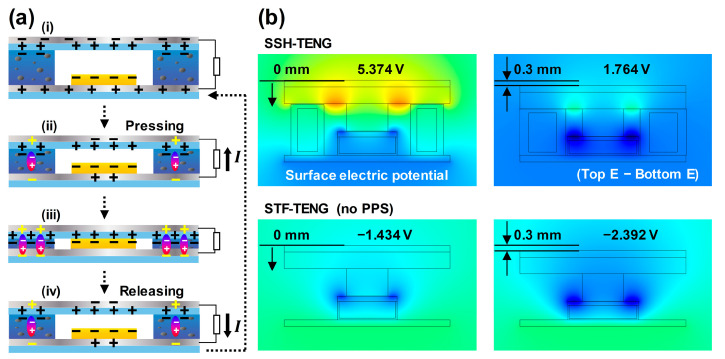
(**a**) Operating principle of the SSH-TENG, showing the (**i**) separated state, (**ii**) contacting state, (**iii**) contact state, and (**iv**) separating state. (**b**) FEM results showing the surface electric potential profile with the contact–separation states.

The working principle of the shear thickening fluid and sponge-hybrid TENG (SSH-TENG) is illustrated in Figure 4a. In the separated state of Figure 4a(i), the electrodes are in a state of equilibrium. With the downward movement of the top layer from applying vertical force, a large flow of electrons from the top electrode to the bottom electrode occurs with electrostatic induction, as shown in Figure 4a(ii). An upward current is generated at the load. The piezoelectric property of the PVDF induces reverse charges at the adjacent electrodes, showing a synergistic effect in the outputs. A slight charge flow can be generated with additional force and with additional polarization due to the piezoelectric effect, creating another equilibrium state, as shown in Figure 4a(iii). By removing the vertical force, the electrons flow from the bottom electrode to the top electrode with the generation of a downward current at the load in Figure 4a(iv). An alternating current is then generated within this cycle of applying and removing the force, followed by the state shown in Figure 4a(i).

In the FEM results, the surface electric potential profile of SSH-TENG and STF-TENG is visualized using color. Between the two electrodes of the SSH-TENG, the potential difference values of 5.374 and 1.764 V were checked in the separated and contact states, respectively. For the STF-TENG, the −1.434 and −2.392 V values were calculated for each separated and contact state. The subtracted potential values from the separated state to the contact state were higher, with an SSH-TENG of 3.61 V compared to that with an STF-TENG of 0.958 V. This enhanced output was due to the increased contact surface of the SSH-TENG compared with that of the STF-TENG.

### 3.4. Electrical Output Optimization for SSH-TENG

**Figure 5:**
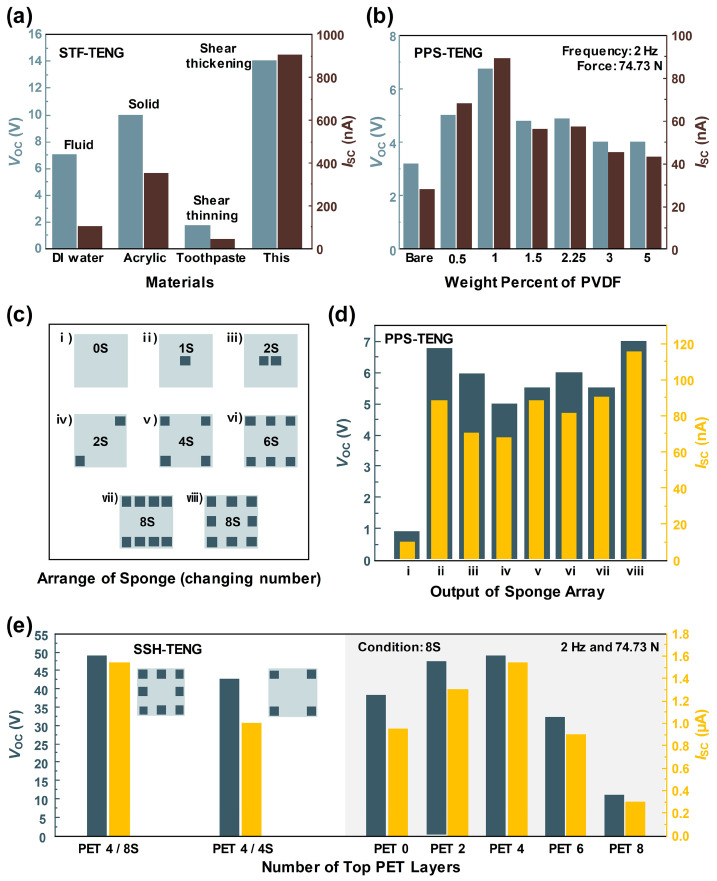
(**a**) Comparison of the four different phases of materials with STF-TENG. (**b**) Electrical output optimization of PPS-TENG, with the wt.% of PVDF in the sugar template. (**c**) Eight different layouts on the sponge for optimizing the PPS-TENG outputs. (**d**) Electrical outputs of PPS-TENG when changing the layouts of the sponge. (**e**) Electrical outputs of the SSH-TENG when changing the thickness of the top PET layers.

The property of the STF with the triboelectric outputs was checked by changing the phase of the inserted materials. Fluid (DI water), solid (acrylic plate), shear thinning fluid (toothpaste), and STF (cornstarch + DI water) were adopted as candidates for the demonstration. A 40% increased output voltage and 157% increased output current were recorded for the STF scenario compared with the solid scenario, due to the solidifying effect and enlarged effective contact area when applying vertical force, as shown in Figure 5a.

The PVDF-PDMS sponge (PPS) was used as the spacer and output-enhancing method in the SSH-TENG. For the optimization of the PPS-TENG output, the wt.% of the PVDF was changed from 0.5 to 5. Mixing the PVDF at concentrations above 5 wt.% affects the capillary action of PDMS, due to the hydrophobicity of PVDF, preventing the formation of a porous structure. Electrical outputs were measured under a vertical input with a force of 74.7 N and a frequency of 2 Hz. After adding the PVDF, showing its piezoelectric property, the triboelectric output was increased due to the polarization of the PVDF when adding pressure. A single sponge was adopted at the center of the electrode area and remained in a contact state during the entire pressing condition. Both electrical outputs in Figure 5b reveal the optimized point at 1 wt.%, producing a *V*_OC_ of 6.75 V and an *I*_SC_ of 89 nA before the agglomeration point was checked with the SEM results. The formation of a porous structure within the PDMS network was also hindered by the agglomeration of the PVDF powder, and the recuperative power of the sponge was decreased.

The arrangement of the multiple sponges was checked in the process of PPS-TENG optimization. The top-view image with the arrangement of PPS is visualized in Figure 5c. After adding the sponge, the triboelectric output was enhanced compared to the 0S (no sponge) scenario in Figure 5d. The highest outputs, with a *V*_OC_ of 7 V and an *I*_SC_ of 115 nA were shown for the 8S model (8 sponges), with the uniformly distributed state seen in case viii, due to the largest contact area and the largest gap between the sponges for the same number of sponges. Case iv showed lower outputs than those of case iii, with the distributed force at the corner and room for the contact in an unbalanced state, even though the number of sponges is the same, at 2 (2S). The lack of sponges at the two corners may induce a nonuniform gap between the two electrodes and hinder effective electrostatic induction.

The outputs of the SSH-TENG were checked when changing the number of sponges and top PET layers with a thickness of 0.3 mm each, as shown in Figure 5e. The number of top PET layers was changed to enlarge the effective contact area between the top layer and the bottom STF layer. The higher outputs with a *V*_OC_ of 48.1 V and *I*_SC_ of 1.54 µA were exhibited in the 8S and PET 4 (4 layers of PET film). When the number of PET layers was reduced, the distance between the PET layer and the STF layer may have increased, consequently reducing the contact area. However, with four or more layers of PET, the repulsive force could have increased, and there was also a decrease in electrical output.

This study rigorously examined the effective contact area by analyzing the electrical output variations associated with changes in the number of PET layers in the SSH-TENG sample. The analytical metrics employed included the slope of the electrical signal generated during contact and the slope of the signal, while the materials remained in contact. To control for the influence of velocity, the output voltage was prioritized as the primary indicator of contact efficacy.

The horizontal slope of the electrical signal, which is indicative of voltage retention during contact, was calculated by dividing the relative voltage drop by the duration of the drop. This relative value of voltage drop was derived from the ratio of the voltage drop to the peak-to-peak voltage. The findings (as shown in the Supplementary Materials) revealed that the horizontal slopes were lower for PET 0 (−0.38 s^−1^ in Figure S1a), 2 (−0.25 s^−1^ in Figure S1b), and 4 (−0.35 s^−1^ in Figure S1c and −0.41 s^−1^ in Figure S1f) compared to PET 6 (−0.56 s^−1^ in Figure S1d) and 8 (−1.06 s^−1^ in Figure S1e). This suggests an optimal distance between the PET and the surface of the solidified STF for PET 0, 2, and 4, which facilitated effective contact-separation. Conversely, the reduced distance in PET 6 and 8 enabled continued force application after contact, transitioning the STF from a solid to a fluid state.

Furthermore, the vertical slope, which measures the rate of voltage increase during contact, was calculated by dividing the voltage increase by the corresponding duration. For PET 0, 2, and 4 with 8S, the slope values were 1.83, 1.79, and 2.09 kV/s, respectively, highlighting the impact of applied pressure on electrical output. Notably, PET 4 with 8S exhibited a 16.8% higher vertical slope compared to PET 4 with 4S (1.79 kV/s), alongside a reduced voltage drop of 0.31 V during contact. The increased number of contact points and the more evenly distributed pressure, facilitated by the additional PPS, helped maintain the contact state in the SSH-TENG.

This detailed analysis emphasizes the utility of electrical signals for determining the state of STF materials within SSH-TENGs, linking physical configurations and applied forces to electrical output characteristics in a measurable and repeatable manner. This approach enables a deeper understanding of the dynamics at play within these sophisticated material systems, providing insights that could guide future improvements in device design and application.

### 3.5. Advanced Electrical Output Characterization of SSH-TENG

**Figure 6:**
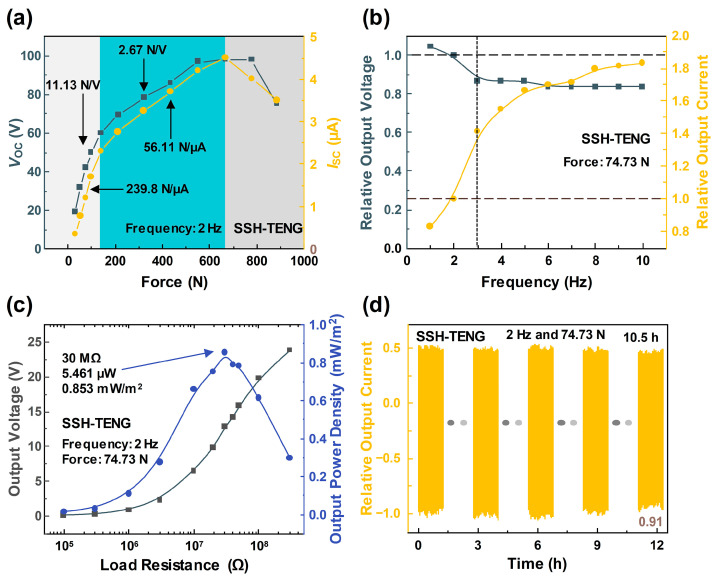
(**a**) Force response of the SSH-TENG. (**b**) Frequency response of the SSH-TENG. (**c**) Output voltage and power, as affected by varying the external load resistor. (**d**) Durability test of the SSH-TENG over 10.5 h.

The electrical outputs of the SSH-TENG were extensively checked, changing the input force and frequency, using the TENG as a motion sensor. In Figure 6a, the electrical outputs were measured within a force range from 30 to 885 N, at 2 Hz. In the lower range under 140 N, the outputs sharply increased with the increased contact force, with a sensitivity of 2.67 N/V and at 56.11 N/µA. In the middle range from 140 to 670 N, the increasing rate of the output values decreased, with a sensitivity of 11.13 N/V and 239.8 N/µA, due to the solidifying state of the STF. The decreasing trends in both electrical outputs were checked over 670 N, due to the repelling force from the additional strong intensity of impacts. The maximum electrical output results were observed with a *V*_OC_ of 98 V and an *I*_SC_ of 4.5 µA, under a mechanical load of 670 N.

The frequency response of the SSH-TENG is displayed in Figure 6b. The frequency was changed from 1 to 10 Hz, with a fixed force of 74.7 N. The output current showed a gradually increasing trend with the term of contact speed, as reported in previous theoretical study results [38]. With the decreased displacement of the electrodynamic shaker head in a low-frequency region under 3 Hz, the output voltage initially decreased with increasing frequency. The voltage remained constant over 3 Hz, due to the fact that open-circuit voltage is affected by surface charge density and gap distance [38].

The power density of the SSH-TENG was also checked to quantitatively check the output level in Figure 6c. By changing the parallel-connected resistor, the output voltage was measured, and the output power density was calculated. The output voltage increased with increasing resistance, up to the highest value in the open-circuit state. Output power was calculated, following Equation (1):

*P* = *V*^2^/*R,* (1)

where *P*, *V*, and *R* indicate the output power, output voltage, and load resistance values, respectively. The power density value was calculated by dividing the output power value by the area of the SSH-TENG, which was 64 cm^2^. The maximum output power density was checked with 0.853 mW/m^2^ at 30 MΩ. According to the maximum power transfer theorem, the internal resistance of the SSH-TENG is the same at 30 MΩ.

The durability of the SSH-TENG sample was confirmed by long-term operation, as shown in Figure 6d. The short-circuit current was continuously measured over 10.5 h, with 74.7 N and 2 Hz of input. The relative output value at the last cycle was 0.91 after 75,600 cycles of contact-separation and this result showed that the SSH-TENG sample can be adopted as a stable motion sensor. Figure S2 in the Supplementary Materials shows that the output current, after being stored for two months under ambient conditions, retained 68.6% of its initial value, demonstrating the operational stability of the device.

### 3.6. Demonstration of the Lying State Detection System with an HTMS Array

**Figure 7:**
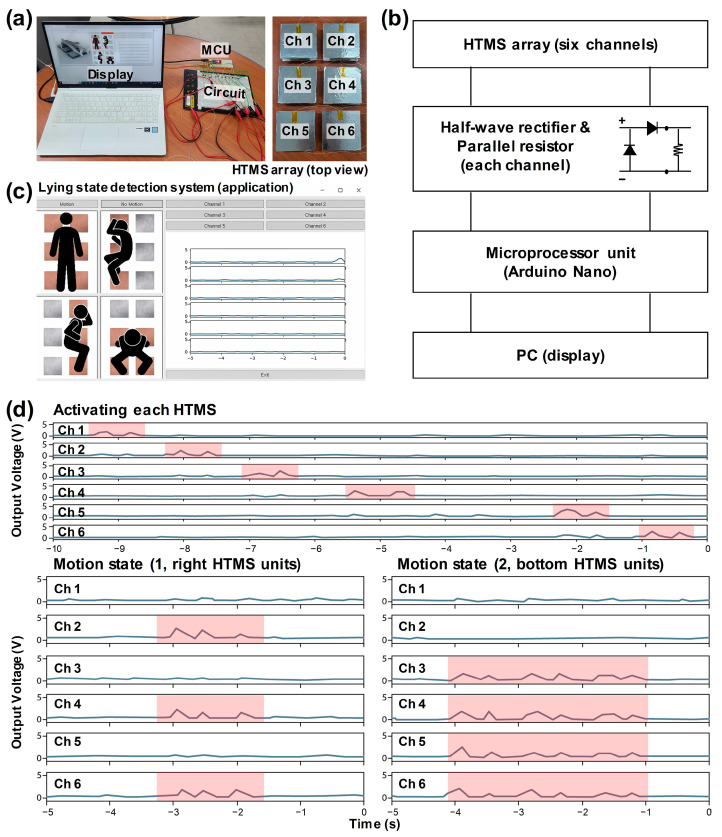
(**a**) Digital camera images of the lying state detection system. (**b**) Block diagram for elucidating the motion detection process. (**c**) Captured application image for setting up alarms for the motion and no-motion states. (**d**) Sensing ability with the HTMS array, showing single and multiple touches.

A lying state detection system was selected to demonstrate the applicability of the SSH-TENG as a hybrid triboelectric motion sensor (HTMS). In this study, 6 SSH-TENGs were used to prepare the 3 × 2 HTMS array, as shown in Figure 7a. Following the connection state model in Figure 7b, each HTMS was connected to a half-wave rectifier and a parallel resistor with 1 MΩ, which was then connected to the microprocessor unit (MCU, Arduino Nano) and PC. The rectification circuit was used to attain a minus current from the MCU. A parallel resistor was adopted to decrease the ghost effect between the adjacent channels and decrease the output voltage level to under 5 V. The attained signals from the HTMS array were displayed with the Python coding shown in Figure 7c. Each channel button blinked when the output level exceeded the threshold value.

The lying state detection system was designed for the early detection of a patient falling from a bed, with turning on one’s side and sitting considered to be dangerous states; detection is achieved by calculating the number of simultaneously blinking buttons. In these aforementioned states, the “Motion” button blinks, as shown in Figure 7c. Moreover, the lying state detection system has the function of preventing bedsores by detecting a no-motion state. The “No Motion” button blinks when there is no movement exceeding the threshold value for any channels during a 10-s period.

A demonstration of the lying state detection system with an HTMS array was simply conducted under simulated conditions, using human hands and arms. The top graph in Figure 7d and in Video S1 in the Supplementary Materials showed the state of pressing each HTMS, one by one, from Ch 1 to Ch 6. The outputs successfully exceeded the threshold value in the entire channel, with a negligible ghost effect, and the channel buttons blinked with the pressing of the corresponding HTMS. The buttons simultaneously blinked when the input force was applied. The motion and no-motion states were checked with one arm and two hands, respectively; the pressing and releasing states are shown in the bottom graphs of Figure 7d and in Video S2 in the Supplementary Materials. The “Motion” and “No Motion” buttons also blinked for each state.

## 4. Conclusions

In this study, an SSH-TENG was developed to implement a lying state detection system based on a motion sensor array. The SSH-TENG comprises a mixture of STF and PVDF-PDMS sponge, maximizing stress during contact-separation phenomena to enhance the output. Experimental findings indicated that the optimized concentration of PVDF was 1 wt.%, resulting in a *V*_OC_ of 6.75 V and an *I*_SC_ of 89 nA, yielding the highest electrical output. Moreover, increasing the quantity of PPS, with eight sponges positioned in the corner and four PET layers on the top, showed a tendency of increased output, with the *V*_OC_ reaching 48.1 V and an *I*_SC_ of 1.54 µA. Investigation into the electrical output and power density results concerning input force and the frequency of the SSH-TENG revealed an increment in output within the force range, albeit at a reduced rate due to STF solidification in the intermediate range, with the highest *V*_OC_ of 98 V and *I*_SC_ of 4.5 µA. An elevation in frequency exhibited a positive correlation with an augmented output current, whereas the output voltage sustained its consistency despite the escalated input frequency. The maximum power density was recorded at 0.853 mW/m^2^ with 30 MΩ. Additionally, the durability of the SSH-TENG sample was confirmed with continuous measurement of the short-circuit current over 10.5 h at 74.7 N and with 2 Hz of input, maintaining consistent output values throughout 75,600 cycles of contact-separation, indicating its viability as a motion sensor. The lying state detection system employed SSH-TENG as a hybrid triboelectric motion sensor (HTMS), utilizing a 3 × 2 array composed of 6 SSH-TENG units connected to the MCU and PC via half-wave rectifiers and 1 MΩ parallel resistors. The HTMS array enables the real-time detection of a patient’s condition, facilitating the early detection of hazardous situations and providing alerts. The durability and performance of the system were validated through various experiments, suggesting promising applications of the proposed SSH-TENG and the lying state detection system in the medical and healthcare industries.

## Data Availability

The raw data supporting the conclusions of this article will be made available by the authors on request.

## Supplementary Materials

The following supporting information can be downloaded at: https://www.mdpi.com/article/10.3390/ma17143536/s1, Figure S1: Output voltage signals with varying numbers of PET layers; Figure S2: Durability performance of output current after 2 months; Video S1: Video demonstrating single-channel output detection; Video S2: Video demonstrating multi-channel motion detection.
