# Supplementary material for: Shear Thickening Fluid and Sponge-Hybrid Triboelectric Nanogenerator for a Motion Sensor Array-Based Lying State Detection System
Source: Materials (Basel). 2024 Jul 17;17(14):3536. doi: 10.3390/ma17143536 (PMC11278460; doi:10.3390/ma17143536)
Supplement: Supplementary file 1 [file materials-17-03536-s001.zip › [20240717] (SM) HTMS.pdf]

Supplementary Materials

Shear Thickening Fluid and Sponge-Hybrid Triboelectric Nanogenerator for Motion Sensor Array Based Lying State Detection System

Youngsu Kim [1], Inkyum Kim [1], Maesoon Im [2,3,4,*] and Daewon Kim [5,6,*]

[1] Department of Electronics and Information Convergence Engineering, Institute for Wearable Convergence Electronics, Kyung Hee University, 1732 Deogyeong-daero, Giheung-gu, Yongin 17104, Republic of Korea
[2] Brain Science Institute, Korea Institute of Science and Technology (KIST), Seoul, 02792, Republic of Korea
[3] Division of Bio-Medical Science and Technology, KIST School, University of Science and Technology (UST), Seoul, 02792, Republic of Korea
[4] KHU-KIST Department of Converging Science and Technology, Kyung Hee University, Seoul, 02447, Republic of Korea
[5] Department of Electronic Engineering, Institute for Wearable Convergence Electronics, Kyung Hee University, 1732 Deogyeong-daero, Giheung-gu, Yongin 17104, Republic of Korea
[6] Center for BrainTechnology, Korea Institute of Science and Technology, 5, Hwarang-ro 14-gil, Seongbuk-gu, Seoul 02792, Republic of Korea
* Correspondence: daewon@khu.ac.kr (D.K.) and maesoon.im@kist.re.kr (M.I.)

Table of contents

1. **Output voltage signals with varying numbers of PET layers**
2. **Durability performance of output current after 2 months**
3. **Video demonstrating single channel output detection**
4. **Video demonstrating multi-channel motion detection**

1. Output voltage signals with varying numbers of PET layers

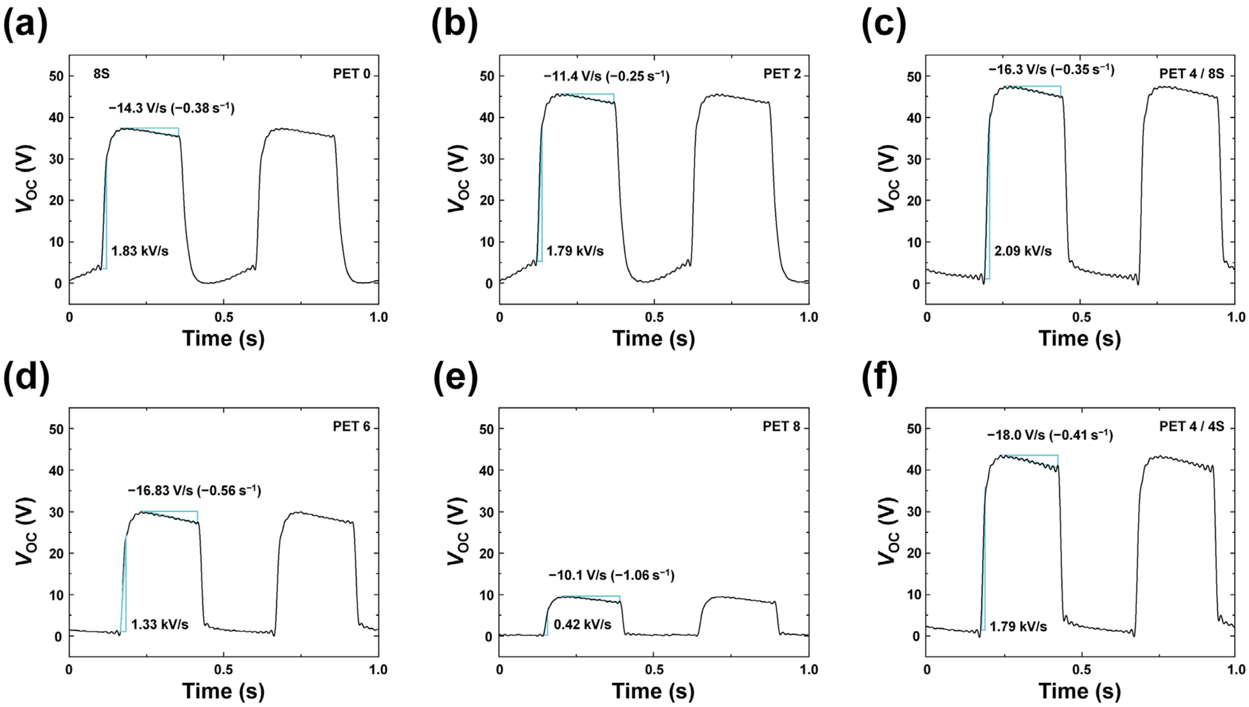

Figure S1. Output voltage signals with changing the numbers of PET: (a) 0, (b) 2, (c) 4 with 8S, (d) 6, (e) 8, and (f) 4 with 4S.

2. Durability performance of output current after 2 months

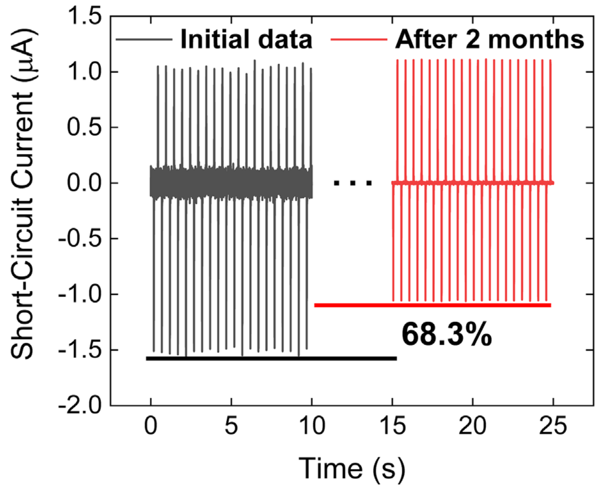

Figure S2. Output current signals comparing the initial data and the data attained after 2 months.

3. Video demonstrating single channel output detection

Video S1. Demonstration of single channel output detection using the HTMS array.

4. Video demonstrating multi-channel motion detection

Video S2. Demonstration of multi-channel output detection using the HTMS array.
